# Hope for primary cardiovascular prevention with the HOPE (Heart Outcomes Prevention Evaluation)-3 trial findings

**DOI:** 10.21542/gcsp.2016.13

**Published:** 2016-06-30

**Authors:** Jassim Al Suwaidi

**Affiliations:** Adult Cardiology, Heart Hospital, Hamad Medical Corporation, Doha, Qatar

## Abstract

The HOPE-3 investigators enrolled 12,705 intermediate-risk participants in 21 countries in a 2-by-2 factorial trial. Subjects were randomized to receive a fixed dose of rosuvastatin or placebo, candesartan plus hydrochlorothiazide daily or placebo, and a third group received combination of antihypertensive and statins versus double placebo. The median follow-up was 5.6 years. The combination of antihypertensive and statin therapy was associated with a significantly lower rate of cardiovascular events than dual placebo. Statin therapy alone was also associated with improved outcome, while antihypertensive therapy had no added benefit compared to placebo.

## Introduction

Cardiovascular disease causes 18 million deaths per year globally and a similar number of non-fatal cardiovascular events^1^. Hypertension is highly prevalent and affects approximately 1 billion adults worldwide^2^. High blood pressure (BP) is also the leading risk factor for cardiovascular disease globally^3^. Although 80% of the global burden of cardiovascular disease occurs in low- and middle-income countries, the majority of trials have been conducted in North America or Europe and involve mainly white persons^1^. There are significant differences between various ethnicities in regards to cardiovascular risk factors and outcome^4^. The HOPE (Heart Outcomes Prevention Evaluation)-3 trial was conducted to study the efficacy of antihypertensive and statin therapies. Participants were of various ethnic backgrounds (six continents), did not have cardiovascular disease, but were at intermediate risk for future cardiovascular events^1,3,5^.

## The Study

The study was conducted in 228 centers in 21 countries. The trial had a 2-by-2 factorial design. The trial evaluated cholesterol lowering with rosuvastatin (10 mg daily), BP-lowering with candesartan (16 mg) mg plus hydrochlorothiazide (12.5 mg) daily, and the combination of both interventions versus placebo for the prevention of cardiovascular events among persons who did not have cardiovascular disease and were at intermediate risk (defined as an annual risk of major events of approximately 1%).

Eligibility criteria included men ≥55 years and women ≥65 years of age who had at least one of the following cardiovascular risk factors: elevated waist-to-hip ratio, history of a low level of high-density lipoprotein cholesterol, current or recent tobacco use, dysglycemia, family history of premature coronary disease, and mild renal dysfunction. (Women ≥60 years of age that had at least two such risk factors were also included). Exclusion criteria included presence of cardiovascular disease and the presence of an indication for or contraindication to statins, angiotensin-receptor blockers (ARB), angiotensin-converting enzyme (ACE) inhibitors, or thiazide diuretics. The trial did not mandate specific lipid or blood pressure levels for entry.

Eligible participants entered a single blind run-in phase, during which they received active treatments (both BP lowering and for cholesterol lowering) for 4 weeks. Participants who adhered to the assigned regimen and who did not have an unacceptable level of adverse events were randomized as follows: one group to receive a fixed combination of candesartan 16 mg and hydrochlorothiazide 12.5 mg daily or placebo, the second was randomized to receive rosuvastatin at a dose of 10 mg daily or placebo, and the third was randomized to receive combined therapy (with rosuvastatin and both antihypertensive agents (3180 participants) or two placebos (3168 participants). Follow-up visits occurred at 6 weeks and 6 months after randomization and every 6 months thereafter. The median follow-up was 5.6 years^1,3,5^.

## Results

The mean age of the participants was 65.7 years, the mean body mass index (BMI) was 27.1. 37.9% reported a history of hypertension and 21.9% were taking antihypertensive agents (other than ARBs, ACE or thiazides) The mean systolic BP was 138.1/81.9 mmHg, the median fasting glucose was 5.3 mmol per liter, and the mean LDL-cholesterol was 3.31 mmol per liter. A total of 46.2% were women. A total 20% of the participants were white, 49.1% were Asian, 27.5% were Hispanic, and 3.3% were black or belonged to another ethnic group. Overall rates of adherence to medications were high.

### Blood pressure lowering versus placebo

There were no significant differences between the active-treatment group and the placebo group in the incidence of the first co-primary outcome (4.1% vs. 4.4%, respectively, HR 0.93, 95% CI 0.79-1.10, p = 0.4) or the second co-primary outcome (4.9% vs. 5.2% respectively, HR 0.95, 95% CI 0.81-1.11, p = 0.51). There were also no significant differences between the groups in the incidence of secondary outcomes and the components of the co-primary outcomes, in total mortality, in the incidence of new-onset diabetes, or in the post hoc outcome of total cardiovascular events. In regards to *safety;* there were no differences between the 2 groups in the rates of cancer, hospitalization for cardiovascular or non-cardiovascular causes, or death from noncardiovascular causes^3^.

### Rosuvastatin versus placebo

The first co-primary outcome occurred in 3.7% in the rosuvastatin group versus 4.8% in the placebo group (HR = 0.76, 95% CI 0.64-0.91; p = 0.002); NNT = 91. The second co-primary outcome occurred in 4.4% of the rosuvastatin group and 5.7% of the placebo group (HR = 0.75, 95% CI 0.64-0.88; p < 0.001); NNT = 73. In regards to safety; a significantly smaller number of participants in the rosuvastatin group than in the placebo group were hospitalized for cardiovascular causes (4.4% vs. 5.8%, p < 0.001). More participants in the rosuvastatin than placebo group had muscle pain or weakness (5.8% vs. 4.7%, p = 0.005), however there no significant differences between the 2 groups in the rate of discontinuation of medications because of muscle weakness or the development of rhabdomyolysis or myopathy. There was no significant difference between the 2 groups in the number of participants who had new-onset diabetes^1^.

### Combination therapy (rosuvastatin and blood pressure lowering agents) versus dual-placebo

The first co-primary outcome occurred in 3.6% in the combined therapy versus 5% in the dual-placebo group (HR = 0.71, 95% CI 0.56-0.90; p = 0.005); NNT = 72. The second co-primary outcome occurred in 4.3% of the combined therapy and 5.9% of the dual-placebo group (HR = 0.75, 95% CI 0.57-0.89; p = 0.003); NNT = 63. There were also significant differences between the 2 groups in the incidence of secondary outcome (4.6% vs. 6.5% respectively, HR 0.71; 95% CI 0.57-0.87, p = 0.001) and the incidence of any kind of stroke (1% vs. 7%, p = 0.009). In regards to *safety;* there were no significant differences between the groups in the rate of new onset diabetes, renal dysfunction, syncope, liver function abnormalities, eye problems or cancer. Although the rates of muscle weakness or pain and of dizziness were higher in the combined-therapy group than in the dual placebo group, these effects were reversible by temporary discontinuation of the trial drug^5^.

## Discussion

HOPE-3 demonstrated the following:

1. Treatment with fixed dose candesartan plus hydrochlorothiazide over a period of 5.6 years lowered blood pressure by 6.0/3.0 mmHg from baseline but did not result in a significantly lower risk of cardiovascular events as compared with placebo. The average BP of participants at baseline was 138.1/81.9 mm Hg; approximately 1/3 of participants had a history of hypertension and approximately 22% were taking antihypertensive agents. As compared with placebo, active treatment was associated with a slightly higher risk of symptomatic hypotension, dizziness and lightheadedness but not syncope, renal dysfunction or other adverse events^2^.
  Recently, two trials evaluated antihypertensive drugs in participants with similar average BP to HOPE-3; the Action to Control Cardiovascular Risk in Diabetes (ACCORD)^6^ and Systolic Blood Pressure Intervention (SPRINT)^2^ trials. ACCORD involved 4733 type-2 diabetic patients who were randomly assigned to intensive therapy (targeting systolic BP <120 mmHg) vs. standard therapy (BP <140 mmHg). The study showed no significant differences between the 2 groups in regards to cardiovascular outcome. SPRINT randomized 9361 non-diabetic participants with increased cardiovascular risk to intensive treatment (systolic BP <120 mmHg) or standard treatment (systolic BP<140 mmHg). In contrast to ACCORD trial findings, intensive treatment in SPRINT resulted in lower rates of fatal and nonfatal major cardiovascular events and death from any cause, albeit with significantly higher rates of some adverse events observed in the intensive-treatment group^2^. It appears that the participants’ risk was much higher in these two trials by design (yearly event rates in the control group of 2.1% in the ACCORD trial and 2.2% in the SPRINT trial vs. 0.8% for the first co-primary outcome and 0.9% for the second co-primary outcome in the HOPE-3 trial). Both ACCORD and SPRINT used complex treat-to-target approaches, which resulted in greater lowering of BP than was observed in HOPE-3 trial, but also resulted in higher rates of adverse events. The HOPE-3 investigators hypothesized that greater reduction in BP might have been more effective as was observed in ACCORD and SPRINT^3^.
  Subanalysis in HOPE-3 demonstrated significant trends towards a lower risk of events with therapy in the subgroup for the upper third of systolic BP (>143.5 mmHg; mean 154.1±8.9 mmHg), when compared top those with lower BP^3^.
2. Treatment with rosuvastatin at a dose of 10 mg daily for a period of 5.6 years among patients with lipid levels within the normal range resulted in a lower risk of cardiovascular events than that of placebo, including the risk of a composite of death from cardiovascular causes, nonfatal myocardial infarction, nonfatal stroke, resuscitated cardiac arrest, revascularization, and heart failure. Treatment with rosuvastatin also resulted in significantly lower risks of stroke and myocardial infarction than those with placebo.
  JUPITER (Justification for the Use of Statins in Prevention: an Intervention Trial Evaluating Rosuvastatin) trial^7^ previously randomized 17,802 patients with elevated levels of C-reactive protein (>2.0 mg per liter) and with LDL cholesterol levels of ≤3.37 mmol/L to rosuvastatin 20 mg daily or placebo. The trial was stopped after a median follow-up of 1.9 years because there was a substantially lower cardiovascular events rate in the active treatment arm compared to placebo. When compared to HOPE 3, the reduction in LDL levels with therapy was larger and the reduction in cardiovascular events was also larger, this difference may in part be attributed to early termination of JUPITER resulting in apparent inflation of the benefits^1^.
3. Combination therapy with rosuvastatin, candesartan and hydrochlorothiazide for a median follow-up of 5.6 years was associated with a significantly lower risk of cardiovascular events than dual placebo. The effects of rosuvastatin in the HOPE-3 trial were independent of blood pressure or lipid levels. There was only one case of rhabdomylosis (in the rosuvastatin plus-placebo group), which was detected clinically, indicating that there is little need for routine blood testing with a combined-treatment strategy.

## What we have learned?

Treatment with fixed dose of candesartan and hydrocholorothiazide was not associated with a lower rate of major cardiovascular events than placebo among persons at intermediate risk who did not have cardiovascular disease. Treatment with fixed dose of rosuvastatin with or without two antihypertensive agents was associated with a significantly lower risk of cardiovascular events than the risk with placebo among intermediate-risk persons without previous cardiovascular disease. The results of HOPE-3 are broadly applicable as approximately half of the participants were women and 80% were nonwhite.

## References

[1] Yusuf S, Bosch J, Dagenais G, Zhu J, Xavier D, Liu L, Pais P, López-Jaramillo P, Leiter LA, Dans A, Avezum A, Piegas LS, Parkhomenko A, Keltai K, Keltai M, Sliwa K, Peters RJ, Held C, Chazova I, Yusoff K, Lewis BS, Jansky P, Khunti K, Toff WD, Reid CM, Varigos J, Sanchez-Vallejo G, McKelvie R, Pogue J, Jung H, Gao P, Diaz R, Lonn E, HOPE-3 Investigators (2016 May 26). Cholesterol lowering in intermediate-risk persons without cardiovascular disease. N Engl J Med.

[2] Wright Jr JT, Williamson JD, Whelton PK, Snyder JK, Sink KM, Rocco MV, Reboussin DM, Rahman M, Oparil S, Lewis CE, Kimmel PL, Johnson KC, Goff JC, Fine LJ, Cutler JA, Cushman WC, Cheung AK, Ambrosius WT, SPRINT Research Group (2015 Nov 26). A randomized trial of intensive versus standard blood-pressure control. N Engl J Med.

[3] Lonn EM, Bosch J, López-Jaramillo P, Zhu J, Liu L, Pais P, Diaz R, Xavier D, Sliwa K, Dans A, Avezum A, Piegas LS, Keltai K, Keltai M, Chazova I, Peters RJ, Held C, Yusoff K, Lewis BS, Jansky P, Parkhomenko A, Khunti K, Toff WD, Reid CM, Varigos J, Leiter LA, Molina DI, McKelvie R, Pogue J, Wilkinson J, Jung H, Dagenais G, Yusuf S, HOPE-3 Investigators (2016 May 26). Blood-pressure lowering in intermediate-risk persons without cardiovascular disease. N Engl J Med.

[4] Ahmed E, Gehani A, El-Menyar A, AlBinAli HA, Singh R, Al Suwaidi J (2014 May). Acute coronary syndrome and ethnicity: observations from the Middle East. Future Cardiol.

[5] Yusuf S, Lonn E, Pais P, Bosch J, López-Jaramillo P, Zhu J, Xavier D, Avezum A, Leiter LA, Piegas LS, Parkhomenko A, Keltai M, Keltai K, Sliwa K, Chazova I, Peters RJ, Held C, Yusoff K, Lewis BS, Jansky P, Khunti K, Toff WD, Reid CM, Varigos J, Accini JL, McKelvie R, Pogue J, Jung H, Liu L, Diaz R, Dans A, Dagenais G, HOPE-3 Investigators (2016 May 26). Blood-pressure and cholesterol lowering in persons without cardiovascular disease. N Engl J Med.

[6] Cushman WC, Evans GW, Byington RP, Goff Jr DC, Grimm Jr RH, Cutler JA, Simons-Morton DG, Basile JN, Corson MA, Probstfield JL, Katz L, Peterson KA, Friedewald WT, Buse JB, Bigger JT, Gerstein HC, Ismail-Beigi F, ACCORD Study Group (2010 Apr 29). Effects of intensive blood-pressure control in type 2 diabetes mellitus. N Engl J Med.

[7] Ridker PM, Danielson E, Fonseca FA, Genest J, Gotto Jr AM, Kastelein JJ, Koenig W, Libby P, Lorenzatti AJ, MacFadyen JG, Nordestgaard BG, Shepherd J, Willerson JT, Glynn RJ, JUPITER Study Group (2008 Nov 20). Rosuvastatin to prevent vascular events in men and women with elevated C-reactive protein. N Engl J Med.

